# Diagnostic and prognostic value of serum S100B in sepsis-associated encephalopathy: A systematic review and meta-analysis

**DOI:** 10.3389/fimmu.2023.1102126

**Published:** 2023-01-27

**Authors:** Jiyun Hu, Shucai Xie, Wenchao Li, Lina Zhang

**Affiliations:** ^1^ Department of Critical Care Medicine, Xiangya Hospital, Central South University, Changsha, China; ^2^ National Clinical Research Center for Geriatric Disorders, Xiangya Hospital, Central South University, Changsha, Hunan, China

**Keywords:** sepsis-associated encephalopathy, biomarker, outcome, meta-analysis, S100B

## Abstract

**Background:**

In sepsis, brain dysfunction is known as Sepsis-associated encephalopathy (SAE), which often results in severe cognitive and neurological sequelae and increases the risk of death. Our systematic review and meta-analysis aimed to explore the diagnostic and prognostic value of serum S100 calcium-binding protein B (S100B) in SAE patients.

**Methods:**

We conducted a systematic search of the databases PubMed, Web of Science, Embase, Cochrane databases, CNKI, VIP, and WFSD from their inception dates until August 20, 2022. A Meta-analysis of the included studies was also performed using Review Manager version 5.4 and Stata16.0.

**Results:**

This meta-analysis included 28 studies with 1401 serum samples from SAE patients and 1591 serum samples from no-encephalopathy septic (NE) patients. The Meta-Analysis showed that individuals with SAE had higher serum S100B level than NE controls (MD, 0.49 [95% CI (0.37)-(0.60), Z =8.29, *P* < 0.00001]), and the baseline level of serum S100B in septic patients with burn was significantly higher than average (1.96 [95% CI (0.92)-(2.99), Z =3.71, P < 0.0002]) In addition, septic patients with favorable outcomes had lower serum S100B levels than those with unfavorable outcomes (MD, -0.35 [95% CI (-0.50)-(-0.20), Z =4.60, *P* < 0.00001]).

**Conclusion:**

Our Meta-Analysis indicates that higher serum S100B level in septic patients are moderately associated with SAE and unfavorable outcomes (The outcomes here mainly refer to the mortality). The serum S100B level may be a useful diagnostic and prognostic biomarker of SAE.

## 1 Introduction

Sepsis is one of the leading causes of death in Intensive Care Unit (ICU) patients who are critically ill. Each year, sepsis affects approximately 49 million people, resulting in 11 million deaths, which accounts for 20% of all deaths worldwide (1). In 2017, World Health Organization (WHO) declared sepsis a global health priority and the greatest unmet medical need of our time (2). Sepsis-associated encephalopathy (SAE) is an underlying brain dysfunction that frequently occurs in the absence of overt infection of the central nervous system (3). Because the complex etiology and pathophysiological pathogenesis of SAE are poorly understood, the clinic lacks specific and effective treatment. As a result, there is a pressing need for an accurate, rapid, and simple test, such as biomarkers, to assess the diagnosis and prognosis of SAE.

Biomarkers are objective indicators used to evaluate the physiological or pathological state and to judge the occurrence, development, and prognosis of diseases. They can reflect characteristic changes that can be measured in the environmental interactions of organisms (4). It is possible to identify, predict, or develop new treatment strategies for SAE by using a biomarker or a panel of biomarkers (5); the small size of miRNAs allows them to pass through the blood-brain barrier (BBB) more easily than other biomolecules in SAE (6); the levels of zonula-occludens (ZO-1) were positively correlated with the APACHE II score, SOFA score as well as lactate levels of SAE patients (7); high mobility group box 1 (HMGB1) mediates cognitive impairment in sepsis survivors, and it may be possible to prevent or reverse cognitive impairments by administering anti-HMGB 1 antibodies (8).

In addition to these biomarkers, the role of S100 calcium-binding protein B (S100B) in the guidance of therapeutic options and surveillance strategies in SAE has also been demonstrated in recent studies (9–11). S100B is a calcium-binding protein, predominantly synthesized in and constitutively secreted by astrocytes, oligodendrocytes of the central nervous system, and Schwann cells of the peripheral nervous system (12, 13). It is mainly present in the cytoplasm in a normal state and regulates protein phosphorylation, cell proliferation and apoptosis, energy metabolism, and inflammatory response through the calcium signaling pathway; in a pathological state, it is mainly secreted into the cell in the form of autocrine and paracrine (14). S100B protein plays a crucial role in Alzheimer’s disease, Parkinson’s disease, multiple sclerosis, Schizophrenia and epilepsy because the high expression of this protein directly targets astrocytes and promotes neuroinflammation (15, 16). Experimental animal studies have revealed that the brain is the primary source of S100B during endotoxemia (17) and play a crucial role in acute brain injury and long-term cognitive impairment during sepsis by regulating mitochondrial dynamics through RAGE/ceramide pathway (18); these findings make S100B a candidate as an essential biomarker of SAE. The purpose of our Systematic Review and Meta-Analysis was to evaluate the potential diagnostic and prognostic value of S100B in SAE patients.

## 2 Methods

### 2.1 Search strategy

All studies published before August 20, 2022, were searched in PubMed, Web of Science, Embase, Cochrane databases, CNKI (China National Knowledge Infrastructure), VIP (China Science and Technology Journal Database) and WFSD (Wanfang Data Knowledge Service Platform). The Medical Subject Heading (Mesh) headings or keywords as: (“S100B,” or “S100 calcium binding protein B,” or “S100,” or “S100-B,” or “S100Beta,” or “S100B,”) AND (“sepsis,” or “severe sepsis,” or “septic shock”). There were no restrictions on language. All cited references were reviewed to identify additional studies.

### 2.2 Inclusion and exclusion criteria

The meta-analysis was limited to studies dealing with the serum S100B in SAE patients. Studies that met the following criteria were identified (1): all patients should meet the confirmed sepsis or septic shock definition, and experiments should be Sepsis-associated encephalopathy patients (SAE), controls should no-encephalopathy septic patients (NE) (2); evaluation of S100B in serum samples. We included both prospective and retrospective studies without restrictions. The exclusion criteria were as follows (1): duplicate publications or other types of patients (2); studies lacking original or complete data (3); animal studies or reviews; and (4) not involving the selected biomarker.

### 2.3 Quality assessment

Two independent reviewers (J-YH and S-CX) performed a study quality assessment. The quality and risk of bias of the selected studies were assessed using the Quality Assessment of Diagnostic Accuracy Studies version 2 (QUADAS-2) assessment tool according to the recommendation by the Cochrane Collaboration (19). We analyzed the following two domains: risk of bias and applicability. Each category had its assessment protocol. We identified the risk of bias in each domain as low, unclear, or high risk based on the methods used to ensure that each form of bias was minimized. Any disagreements were discussed and resolved by the entire review team.

### 2.4 Data extraction

Three reviewers independently extracted data from each included study according to the selection criteria. After extraction, data were reviewed and compared by the first author. Disagreements were resolved by consensus. The data extracted included study characteristics (first author and year), participant characteristics (age, sex ratio, and sample size), and methodological characteristics (assay, cutoff and collection time, and clinical trial design types). We unified serum S100B levels to (ng/mL). Additional information can be obtained by directly questioning the primary authors when possible to acquire and verify the data. If there were several serum S100B collection time points in one study, we marked them separately at different time points, such as Feng 2017(1d) and Feng 2017(3d).

### 2.5 Statistical analysis

Parametric variables were described as means and SDs, and nonparametric variables were described as medians and interquartile ranges (IQRs). If mean data were not reported, we used the method by Wan et al. to estimate the mean and SD using the median and IQR or median and range to estimate the mean and SD (20, 21). I-squared (*I^2^*) statistics and Q test were used to evaluate the effect of study heterogeneity on the Meta-Analysis results (22). According to the Cochrane review guidelines, if severe heterogeneity was present at *P*<0.1 or *I^2^* >50%, a random-effects model was selected; otherwise, a fixed-effects model was used. Moreover, Subgroup analyses were performed according to serum sample collection time or serum S100B measurement assay. Potential publication bias was assessed using Begg’s and Egger’s tests and funnel plots. A sensitivity analysis was performed to determine the stability and consistency of the meta-analysis results. All analyses were performed using Review Manager version 5.4 (RevMan, The Cochrane Collaboration, Copenhagen) and STATA software (version 16.0, StataCorp, College Station, TX). For all analyses, *P*<0.05 was considered significant. For publication bias, *P*>0.1 was considered significant.

## 3 Results

### 3.1 Search results

**Figure 1:**
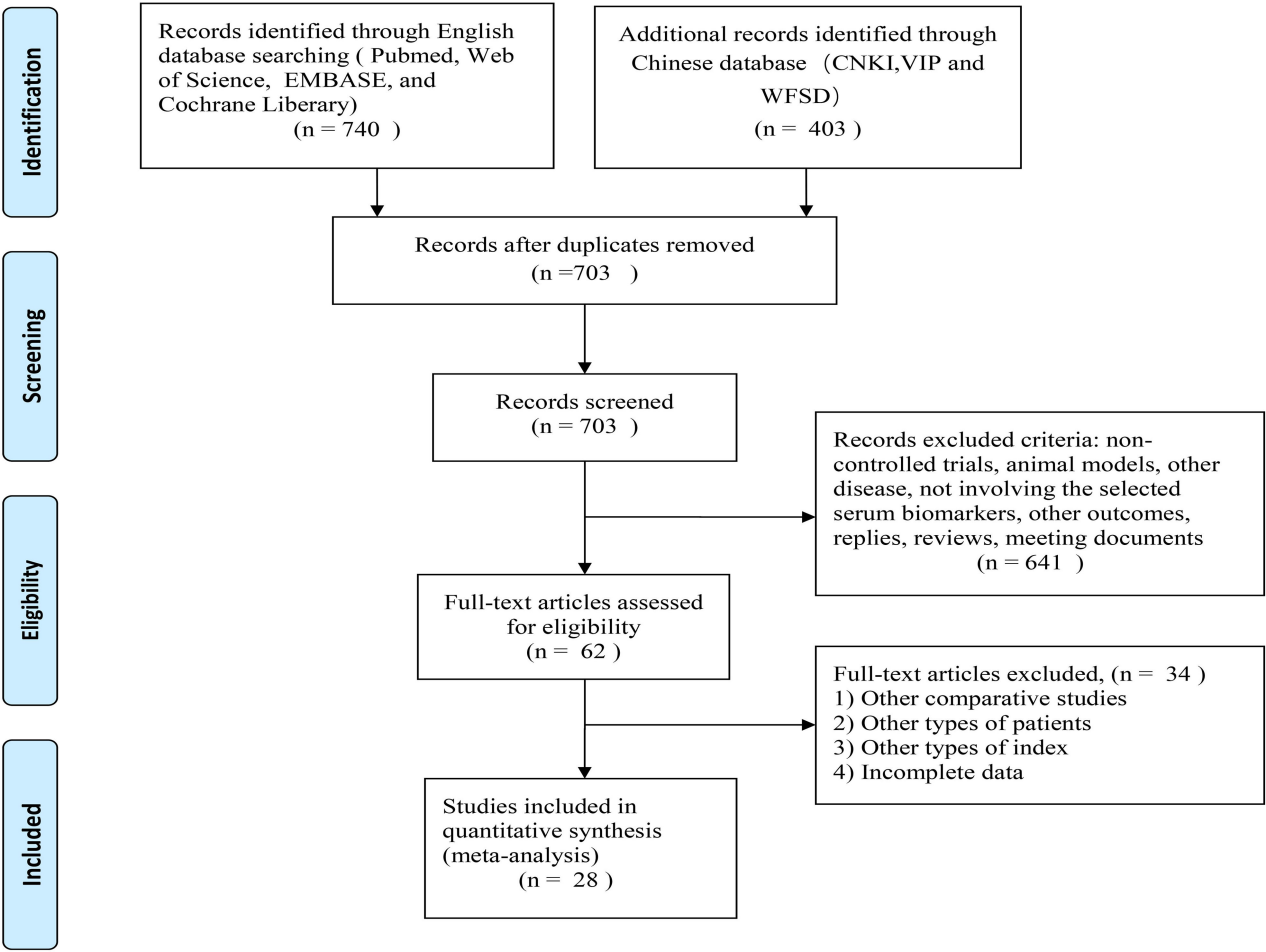
Literature search strategy and screening process.

**Figure 1** illustrates the study selection process. There were 1143 relevant studies under the search words (PubMed 137, Web of Science 205, EMBASE 390, Cochrane Library 5, CNKI 27, WFSD 350, VIP 26), of which 440 were excluded due to duplicates. A total of 703 studies were identified through a literature search and screening of titles and/or abstracts. Of these, 641 studies were unrelated to the topic and excluded. After the assessment of eligibility using full text, 62 studies were excluded. Finally, 28 studies met all inclusion criteria and were included in the meta-analysis: 12 studies from the English database (23–34); 16 studies from the Chinese database (35–50). The selected details of the individual studies are listed in **Table 1**. In the current meta-analysis, there were 1401 serum samples from patients with SAE and 1591 serum samples from septic patients without encephalopathy.

**Table 1:** Specific basic characteristics of the included studies (SAE, Sepsis-associated encephalopathy patients; NE, no−encephalopathy septic patients; NA, not announce; h, hour; d, day; m, month; ELISA, enzyme-linked immunosorbent assay; ECLIA, electrochemiluminescence immunoassay; CLIA, chemiluminescence immunoassay; ICA, immunochromatography assay; FICA, fluorescence immunochromatography assay; RIA, radioimmunoassay; WB, western blotting).

study and year	SAE sample size (Males/Female)	NE sample size (Males/Female)	Age	Sample Collection time	Assay	SAE S100B cutoff (ng/mL)	Design
**Chen 2019 (** 45)	42(-) All patients: 100(56/44)	58 (–)	68 ± 5.4	ICU admission	ELISA	0.53 ± 0.28	NA
**Cui 2022 (** 46)	79(45/34)	121(70/51)	SAE:72.78 ± 4.01 NE:72_86 ± 4.60	Within 48 h	FICA	0.53 ± 0.09	Retrospective study
**Erikson 2019 (** 29)	10(4/6)	12(10/2)	SAE: 62.4 (49‐70.5) NE: 61.8 (60.1‐78.5)	When CAM‐ICU assessed	CLIA	0.30 (0.19‐0.59)	Prospective observational study
**Feng 2017 (** 31)	36(21/15)	23(14/9)	SAE:52 ± 14 NE:57 ± 15	1,3 d	CLIA	1d:0.33(0.15,0.54) 3d:0.19(0.10,0.29)	Retrospective study
**Guo 2021 (** 26)	30(17/13)	90(42/48)	SAE:57.61 ± 4.16 NE:56.91 ± 4.85	NA	ELISA	0.27 ± 0.06	NA
**Hamed 2009 (** 25)	16(-) All patients: 40(24/16)	24(-)	51.75 ± 4.09 months	NA	ELISA	0. 24 ± 0.07	NA
**Hu 2020 (** 47)	40(-)	40(-)	NA	1 h,3 d,5 d	ELISA	1h:0.50351 ± 0.41551 3d:0.36315 ± 0.2466 5d:0.0683 ± 0.02235	NA
**Jiang 2021 (** 35)	26(18/8)	38(27/11)	SAE:42.45 ± 3.48 NE:41.2 ± 3.5	4 h	ELISA	0.16446 ± 0.02921	Retrospective study
**Kang 2022 (** 40)	22(14/8)	25(14/11)	SAE:27.5(11.3-54.5)months NE:21.0(9.0-32.5)months	Within 24 h	ELISA	1.8 ± 0.2	Retrospective study
**Li 2019 (** 42)	28(-) All patients: 100(56/44)	102(-)	58.6 ± 6.7	1 d	ELISA	0.92 ± 0.15	Retrospective study
**Li 2022 (** 23)	21(13/8)	20(12/8)	SAE: 37 ± 5 NE: 38 ± 4	12,24,48 h	NA	12h:2.38 ± 0.21 24h:3.52 ± 0.16 48h:2.45 ± 0.18	Retrospective study
**Liao 2017 (** 37)	28(20/8)	10(8/2)	SAE: 55 ± 13 NE: 51 ± 16	1 h,3 d	ELISA	1h: 0.5 ± 0.24 3d:0.58 ± 0.33	NA
**Lu 2016 (** 27)	34(24/10)	52(33/19)	SAE: 59.15 ± 8.8 NE: 58.39 ± 8.14	NA	NA	1.21 ± 0.15	Retrospective study
**Nguyen 2014 (** 33)	107 (-) All patients: 128(83/45)	21(-)	65 ± 14	ICU admission,4 d	RIA	ICU admission:0.13 (0.06, 0.49) 4d:0.12 (0.08, 0.24)	Prospective observational study
**Pfister 2008 (** 34)	All patients: 16(10/6)	NA	73.08 ± 8.78	NA	CLIA	NA	NA
**Wang 2019 (** 48)	48(20/28)	12(7/5)	SAE: 55 ± 13 NE: 56 ± 7	1 h,3 d	ELISA	NA	NA
**Wang 2020 (** 41)	30(17/13)	30(19/11)	SAE: 50.5 ± 2.3 NE: 50.8 ± 2.5	1 d	WB	0.28 ± 0.04	NA
**Wang 2022 (** 49)	45(29/16)	35(22/13)	SAE:55.42 ± 14.63 NE: 56.37 ± 15.74	1 d,3 d	ELISA	1d:0.32(0.162, 0.579) 3d:0.18(0.116, 0.307)	NA
**Wu 2020 (** 30)	59(38/21)	45(32/13)	SAE: 54 ± 15 NE: 58 ± 14	1,3d	ECLIA	1d:0.291(0.174–0.478) 3d:0.226(0.129–0.447)	Prospective and cohort study
**Yan 2019 (** 28)	58(44/14)	94(60/34)	SAE: 55.8 ± 16.4 NE: 55.0 ± 18.3	Within 24 h	ELISA	0.5(0.3, 1.3)	Retrospective study
**Yao 2014 (** 24)	48(33/15)	64(40/24)	SAE: 56 ± 16 NE: 52 ± 17	1 d	ECLIA	0.306 (0.157,0.880)	Prospective observational study
**Yu 2020 (** 39)	90(49/41)	90(47/43)	SAE: 53.61 ± 12.74 NE: 52.89 ± 11.65	NA	ELISA	0.96 ± 0.14	NA
**Yu 2022 (** 38)	67(37/30)	95(51/44)	SAE:70.3 ± 8.3 NE: 69.7 ± 8.6	NA	ICA	1.03 ± 0.32	Retrospective study
**Zhang 2015 (** 36)	38(24/14)	36(22/14)	SAE:56 ± 17 NE: 54 ± 15	1 d	ELISA	1.81 ± 0.22	Prospective study
**Zhang 2016 (** 32)	29(20/9)	28(13/15)	SAE:55.55 ± 12.72 NE: 56.21 ± 12.85	Within 24 h	ELISA	2.50 ± 0.49	Prospective observational study
**Zhao 2016 (** 44)	56(30/26)	60(32/28)	SAE:47 ± 13.4 NE: 49 ± 13.2	ICU admission	ELISA	0.775 ± 0.356	NA
**Zhao 2020 (** 43)	22 (–) All patients: 100(58/42)	78 (–)	65.3 ± 12.1	1 d	ELISA	0.92 ± 0.11	NA
**Zhao 2022 (** 50)	28(16/12)	32(18/14)	SAE:55.89 ± 16.55 NE: 55.23 ± 16.71	NA	ELISA	0.99 ± 0.28	NA

### 3.2 Quality assessment

**Figure 2:**
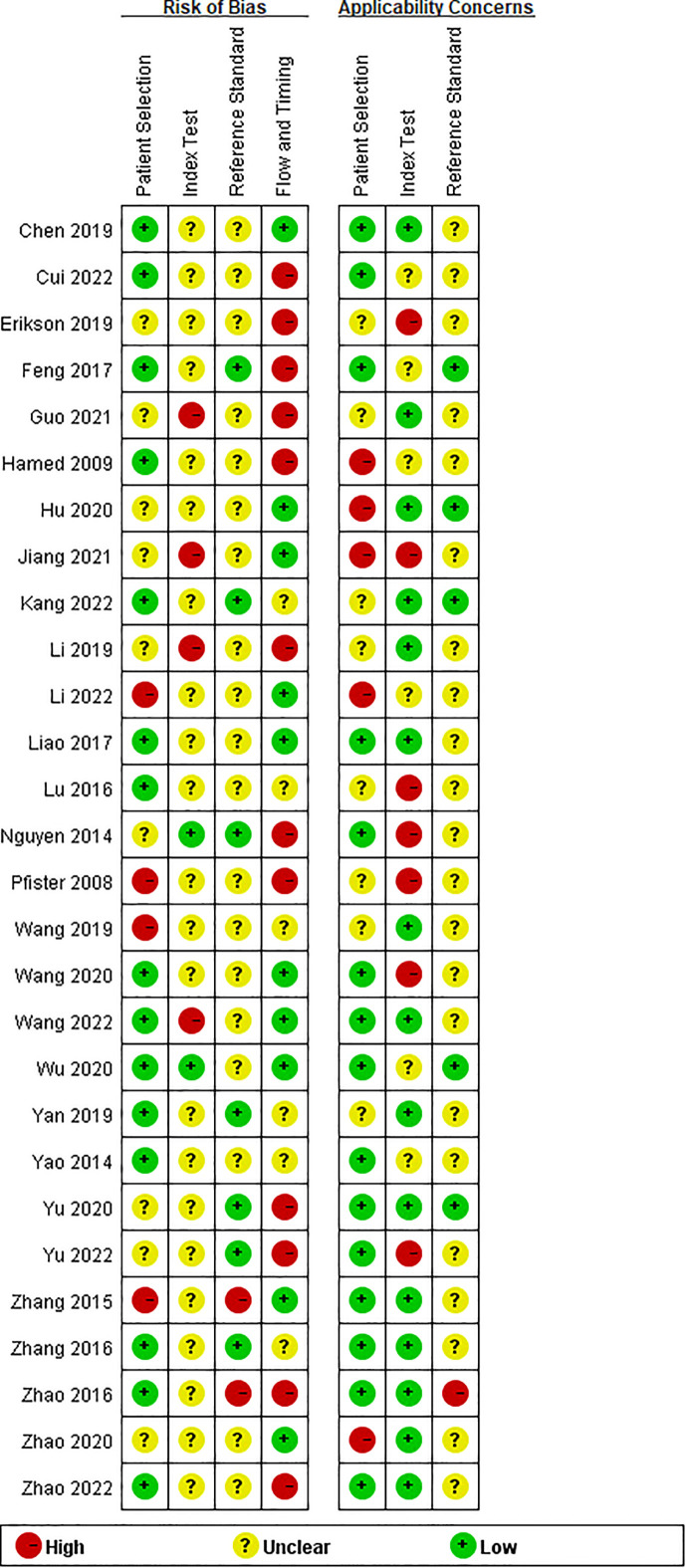
Risk of bias and applicability concerns summary: review authors’ judgements about each domain for each included study.

**Figure 3:**
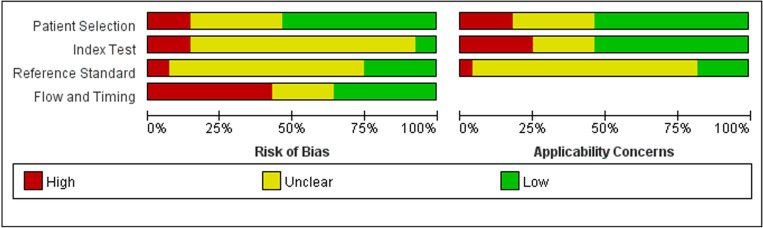
Risk of bias and applicability concerns graph: review authors’ judgements about each domain presented as percentages across included studies.

Twenty-eight studies examined the risk of bias and applicability concerns using a modified QUADAS-2 assessment tool, as displayed in **Figures 2** and **3**. There were 15 studies with a low risk of patient selection, two studies with low risk, and 22 with unclear risk on index test; seven studies had low risk on the reference standard; ten studies had a low risk of flow and timing. There were 15 studies with low concern regarding patient selection, 14 with low concern about the index test, and 5 with low concern about the reference standard. In short, high-risk was mainly focused on index tests, flow, and timing items.

### 3.3 Meta-analysis results

#### 3.3.1 Comparison of serum S100B levels between SAE and NE

**Figure 4:**
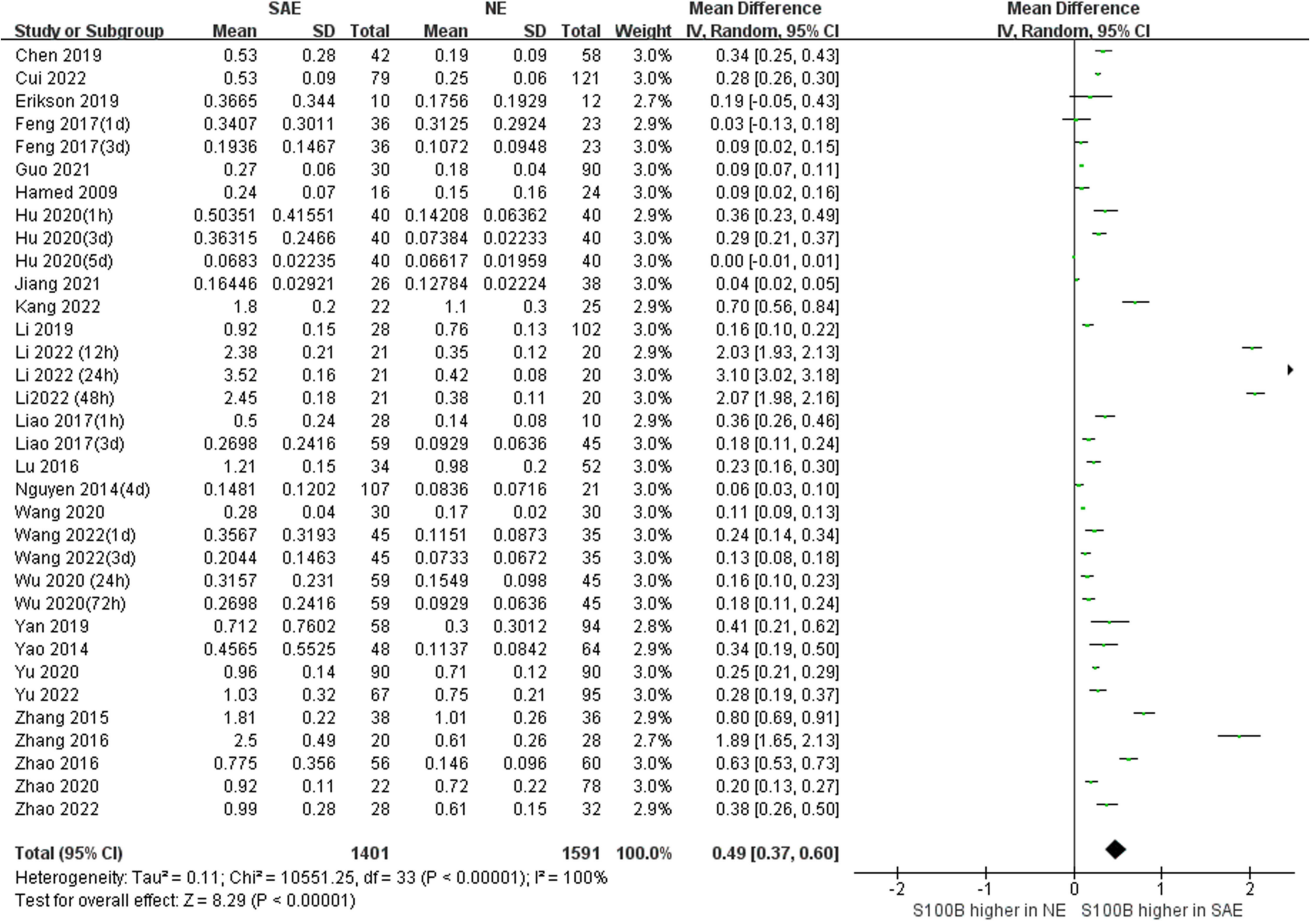
Meta-Analysis forest plot: association between serum S100B level and patients with SAE.

Because we unified serum S100B units to (ng/mL), the Mean Difference (MD) rather than Standardized Mean Difference (SMD) was used to estimate serum S100B levels in both groups. The results of the pooled MD analysis are revealed in **Figure 4**. The heterogeneity test demonstrated significant differences among studies (Chi^2^ = 10551.25, *I^2^* = 100%, *P* < 0.00001); therefore, the random-effects model was applied. The pooled MD was 0.49 (95% CI = 0.37 ~ 0.60, *P* < 0.00001), suggesting that the serum S100B levels in SAE group were significantly higher than levels observed in NE group. Serum S100B may serve as a blood-based biomarker to assist clinical diagnosis and monitor the progression of SAE contusion, which may provide a simple and effective reference basis for clinical treatment decisions.

#### 3.3.2 Comparison of serum S100B levels between favorable outcomes and unfavorable outcomes

**Figure 5:**
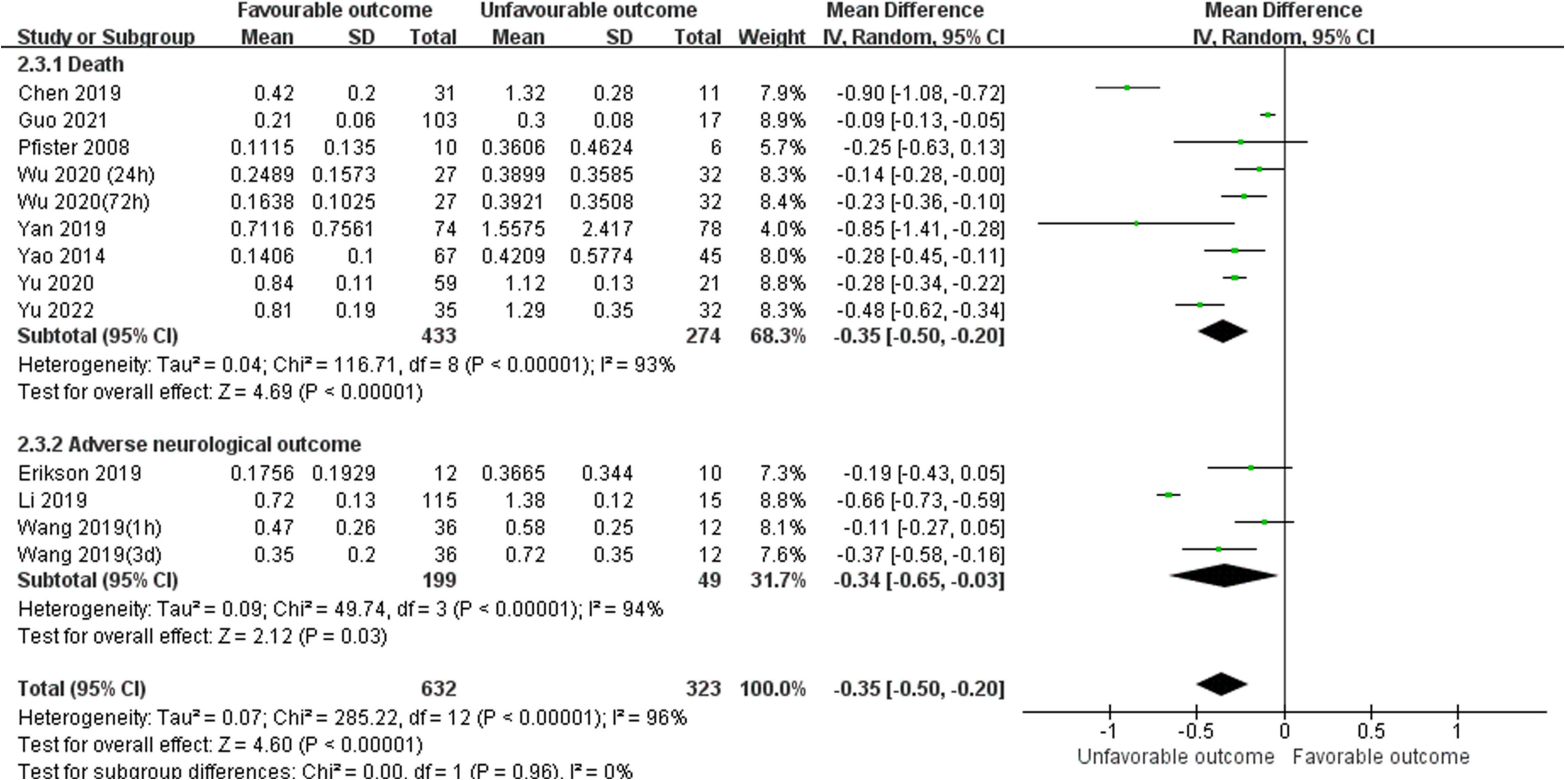
Meta-Analysis forest plot: MDs of serum S100B levels between Favorable outcomes and Unfavorable outcomes.

Unfavorable outcome is defined as death or adverse neurological outcome assessment. There were total twelve studies reported serum S100B levels between favorable and unfavorable outcomes, included eight studies (24, 26, 28, 30, 34, 38, 39, 45) reported death as a primary outcome (MD, -0.35 [95% CI (-0.50)-(-0.20), Z =4.69, *P* < 0.00001]), and three studies (29, 42, 48) reported adverse neurological outcome as a primary outcome (MD, -0.34 [95% CI (-0.65)-(- 0. 03), Z =2.12, *P* =0.03]).The total pooled MD was -0.35 [95% CI (-0.50)-(-0.20), Z =4.60, *P* < 0.00001] which in **Figure 5**, showed that serum S100B levels in unfavorable outcomes group were significantly higher than in favorable outcomes group, so it may be predictive of poorer prognosis for septic patients to have higher serum levels of S100B. The results developed a new biomarker for predicting clinical status and malignant potential of sepsis that warrants further investigation as a predictive biomarker.

### 3.4 Subgroup analysis

**Figure 6:**
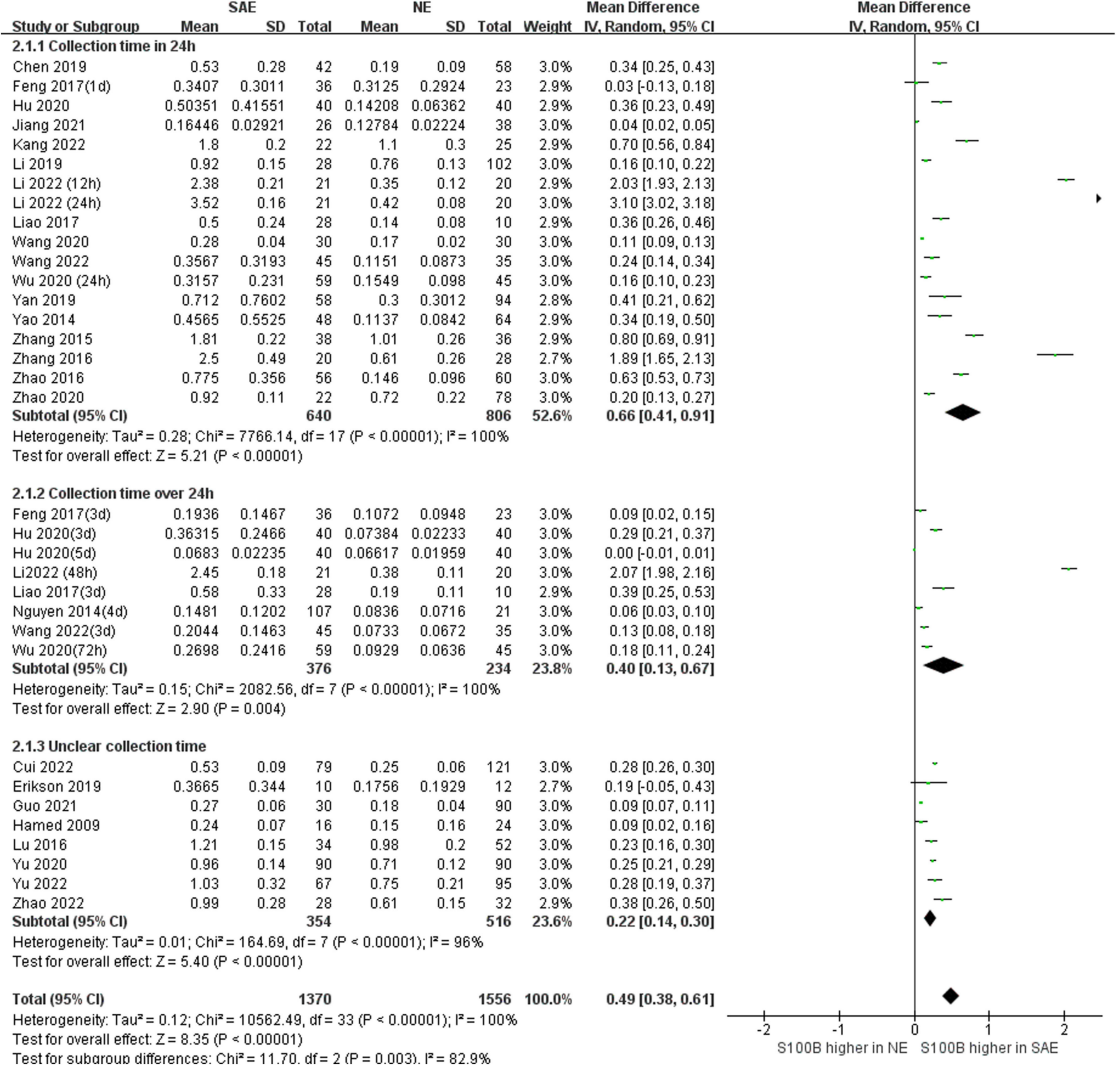
Meta-Analysis forest plot: association between serum S100B level and patients with SAE in different serum sample collection time after ICU admission.

**Figure 7:**
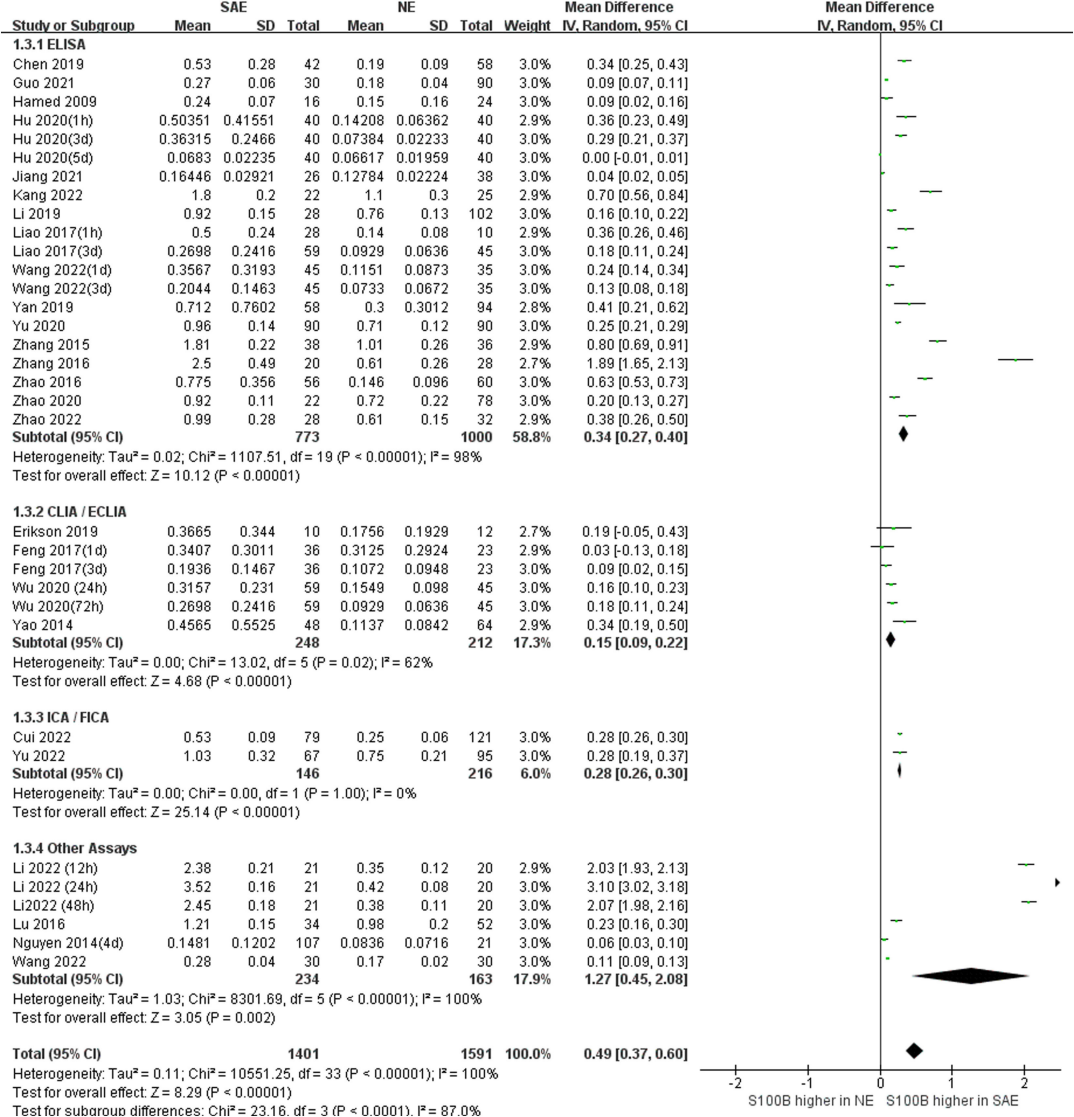
Meta-Analysis forest plot: association between serum S100B level and patients with SAE in different measure assay.

Subgroup analysis was performed to explore the impact of different serum sample collection times after ICU admission (smaller than or equal to 24 h, greater than 24 h, and unclear time) and different S100B measurement assays (ELISA, CLIA/ECLIA, ICA/FICA, and other assays) which displayed in **Figure 6** and **Figure 7**. The subgroup analysis results suggested that different serum sample collection times (collection time ≤ 24h:*I^2^ =*100%, *P*<0.00001; collection time>24h:*I^2^ =*100%, *P*<0.00001; unclear collection time: *I^2^ =*96%, *P*<0.00001) and measurement assay (ELISA: *I^2^ =*98%, *P*<0.00001; CLIA/ECLIA: *I^2^ =*62%, *P*=0.02; ICA/FICA: *I^2^ =*0%, *P*=1.00; Other assays: *I^2^ =*100%, *P*<0.00001) were not sources of heterogeneity. Furthermore, the combined results of subgroup analyses were statistically significant and consistent with the overall combined results, showing serum S100B levels in the SAE group were significantly higher than levels in NE control group whether different serum sample collection time points (MD, 0.49 [95% CI (0.38)-(0.61), Z =8.35, *P* < 0.00001]) or different measure assays (MD, 0.49 [95% CI (0.37)-(0.60), Z =8.29, *P* < 0.00001]) were used. Specific range of serum S100B concentrations in SAE are discussed in the Discussion section.

### 3.5 Publication bias and sensitivity analysis

**Figure 8:**
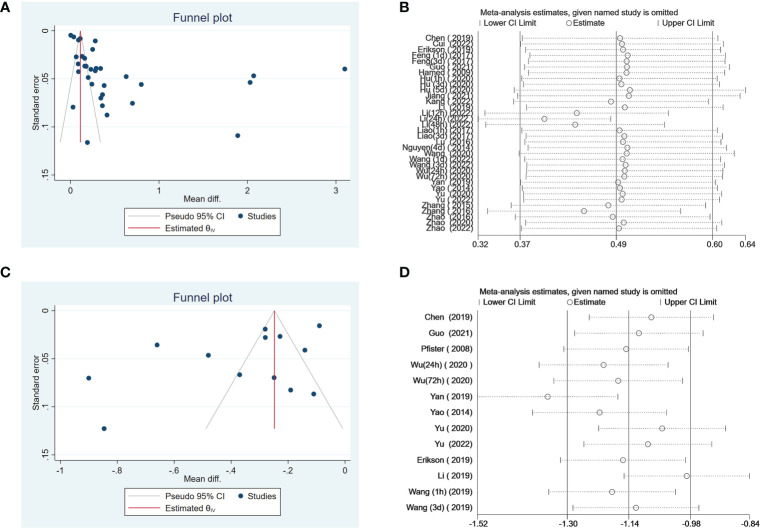
Funnel plot showed that it was asymmetry, suggesting potential publication bias may exist in association between serum S100B level and SAE **(A)**, association between serum S100B level and outcomes of septic patients **(C)**. Effect of individual studies on the pooled MD for the effect of association between serum S100B level and SAE **(B)**, and the effect of association between serum S100B level and outcomes of septic patients **(D)**.

We used Egger’s and Begg’s regression tests and funneled plots to assess the potential publication bias in included studies. When valuated the association between serum S100B levels and SAE, Egger’s (t = 1.49, *P* = 0.1361) and Begg’s tests (Z = 2.45, *P* = 0.0144) of the effective rate indicated publication bias in this included literature. As the association between serum S100B levels and outcomes of septic patients, Egger’s test (t = -1.46, *P* = 0.1444) and Begg’s test (Z = -0.55, *P* = 1.417) of the effective rate also indicated publication bias. Evidence of publication bias was obtained through the visual distribution of funnel plot (**Figures 8A, C**), and the results of the sensitivity analysis indicated that no individual study dominated the results except Yan 2019, and the findings of this meta-analysis were statistically stable (**Figures 8B, D**), but this had to be interpreted with caution when explain prognostic value of S100B due to Yan 2019 had a stronger impact on the overall effect size which in **Figure 8D**.

## 4 Discussion

This meta-analysis aimed to explore the diagnostic and prognostic value of S100B in SAE patients. SAE are frequently encountered in critically ill patients in the ICU and up to 70% of patients with severe systemic infection. Clinically, SAE is characterized by behavioral, cognitive, awakening, and consciousness changes, and a considerable proportion of patients have long-term cognitive dysfunction, which seriously affects daily life and increases the risk of death (51). However, in the absence of an unambiguous definition of SAE and highly accurate diagnostic tools, ICU physicians rely on their own clinical skill set and experience to diagnose SAE.

Our findings demonstrated that serum S100B levels in the SAE group were significantly higher than those observed in the NE group, and serum S100B levels in the favorable outcome group were significantly lower than those in the unfavorable outcome group. The combination of serum S100B and Electroencephalogram (EEG), Computed Tomography (CT), Magnetic resonance imaging (MRI), Transcranial Doppler (TCD) can further confirm the diagnosis and prognosis as well as guide treatment.

A prospective study found that serum S100B is a better biomarker than neuron-specific enolase (NSE) in SAE. GCS scores were related more closely to S100B than NSE, and the area under the curve (AUC) for S100B for diagnosing SAE (*AUC*= 0.824 vs. *AUC*= 0.664) and predicting hospital mortality (*AUC*= 0.730 vs. *AUC*= 0.590) was larger than that for NSE (24). Pfister et al. found a significant association between elevated S100B and sepsis-associated delirium (34); Cohen et al. found S100B can be useful to determine the prognosis for persistent cognitive dysfunction, which defined as the state of altered mentation (AMS) and long-term survival among sepsis patients (52); Calsavara et al. suggested that serum S100B level may associate with anxiety, depression and post-traumatic stress disorder (PTSD) symptoms in sepsis survivors (53). From a clinical perspective, serum S100B provide helpful information for the clinical decision-making and may be a functional biomarker for monitoring clinical conditions for SAE diagnosis and prognosis.

The question from this study should be considered as the range of serum S100B concentrations employed has varied widely across studies, due to differential serum sample collection time or measurement assay or types of patients enrolled in the studies. Serum collection time in our meta-analysis included obscure time points such as ICU admission or within 24 h, and well-defined time points such as days 1, 3, and 7. Subgroup analysis found serum S100B levels which collection time ≤ 24h was higher than > 24h (MD, 0.66 [95% CI (0.41)-(0.91), Z = 5.21, *P* < 0.00001] vs. 0.40 [MD, 95% CI (0.13)-(0.67), Z =2.90, *P* < 0.004]). Thus, S100B may be an early biomarker for SAE which consistent with our previous clinical studies and animal experiments by our group. And patients admitted to the ICU are unpredictable, and emergency and serum collection time may not be sufficiently precise, standardization and generality of its test results remain to be further standardized.

We also explored two special population: Child and burn patients, there are three studies (25, 40, 47) used child participants, and two studies (23, 50) used burn participants. studies from children (MD, 0.28 [95% CI (0.08) -(0.48), Z =2.77, *P* = 0.006]), showing that serum S100B levels in SAE group were significantly higher than those in the NE group whether in adults or children, so there was no age limit for the diagnostic value of serum S100B in SAE (**Figure S1**). The heterogeneity test depicted significant differences among studies from burn (Chi^2^ = 1547.26, *I^2^* = 100%, *P* < 0.00001), so the random-effects model was applied with a pooled (MD, 1.96 [95% CI (0.92) -(2.99), Z =3.71, *P* = 0.0002]), showing that serum S100B levels in SAE patients with burn were also higher than those in NE patients with burn (**Figure S2**), and its levels were significantly above average level (1.96 [95% CI (0.92)-(2.99), Z =3.71, *P* < 0.0002] vs. 0.49 [95% CI (0.37)-(0.60), Z =8.29, *P* < 0.0002]). It is possible that burn sepsis is quite different from mainstream sepsis because of special hypermetabolic reactions and abnormal immune status. Burn stress leads to increased body temperature and heart rate, and immune disorders lead to increased indicators of infection, which is easy to misdiagnose as sepsis. Its pathogenesis is complex, and the interaction of multiple factors may lead to its occurrence (54, 55).

Our results suggest that elevated serum S100B levels are associated with poor prognosis, which also indicates that S100B can be considered an interesting factor for the prevention and treatment of sepsis. Therapies targeting S100B may be promising pharmacological targets to prevent SAE, which may assist in choosing the best combined or primary treatment method. Arundic acid can inhibit the enlargement of brain damage by preventing inflammatory changes caused by the overproduction of S100B protein in astrocytes (56, 57). Moreover, sepsis animal model induced by cecal ligation and perforation (CLP) demonstrated that 10 μg/kg of monoclonal antibody (Anti-S100B) administered intracerebroventricularly could recover habitual memory in the open field task and improve cognitive function (58). The antiprotozoal drug pentamidine can block the S100B/RAGE/NF-κB signaling pathway and reduce neuroinflammation in CLP mouse hippocampus (59), and the mitochondrial division inhibitor Mdivi-1 can inhibit S100B release into plasma in a lipopolysaccharide-induced SAE animal model (60). At present, related antagonistic S100B studies are mostly conducted in animal models and relatively few in clinical applications, and more clinical studies are still needed to prove its therapeutic value for SAE patients in the future.

However, the diagnostic and prognostic role of S100B in SAE is still controversial, and different studies have resulted in large gaps and even opposite conclusions (11, 61), Vuceljic et al. depicted that S100B protein is not a good early predictor of severe sepsis outcomes (62). Weigand et al. suggested no significant difference in serum S100 was observed between sepsis survivors and non-survivors (63). Ehler et al. demonstrated that cerebrospinal fluid S100B levels were not significantly different between sepsis patients and controls (64). Ripper et al. found elevated serum S100B level in patients with delirium but also in septic patients without delirium; this increase was not associated with mortality (65). Considerable variations can be found according to different diagnostic criteria of SAE (66–68), and studies in different eras had different criteria, which may influence the accuracy of the data that we collected. The combination of multiple biomarkers may provide a more objective and reliable guide for the diagnosis and prognosis of SAE, which may be confirmed in further clinical studies.

The specific molecular mechanisms underlying the activity of S100B in SAE have not been elucidated, and it is still unknown whether S100B is the initiating factor or effector of SAE, which means that S100B increased, causing SAE, or SAE causing S100B to increase. Zhang et al. found that S100B regulates mitochondrial dynamics through the RAGE/ceramide pathway, as well as acute brain injury and long-term cognitive impairment during SAE (18). Tsoporis et al. suggested that interaction of RAGE and its ligand S100B after myocardial infarction may play a role in myocyte apoptosis by activating ERK1/2 and p53 signaling (14). Therefore, the role of S100B requires further investigation to produce a clear conclusion on the effects of S100B on SAE.

Except for routine serum or plasma S100B testing, using saliva or urine to detect S100B is simpler and faster, bringing hope for the large-scale promotion of S100B screening. S100B can be extracted simultaneously from serum, urine, cerebrospinal fluid and saliva; two studies revealed that measuring salivary (69) or urine (70) S100B could in place of serum S100B in the diagnosis of TBI. This could avoid the risk of infection from blood tests and reduce the time or equipment required to separate blood components. Larger and confirmatory trials are needed to define salivary or urine biomarker kinetics concerning SAE.

In addition to its role in SAE, S100B has been associated with a variety of neurocritical diseases, such as Traumatic Brain Injury (TBI) (71), aneurysmal subarachnoid hemorrhage (72), acute ischemic stroke (73), and neonatal hypoxic-ischemic encephalopathy (74). In the current COVID-19 pandemic era, most SARS-Coronavirus-2-infected patients admitted to the ICU showed common features of sepsis disease, such as the overwhelmed systemic inflammatory response (75, 76), and a recent prospective study demonstrated that serum S100B of Covid patients is correlated with disease severity, and increased serum levels of S100B correlate with the severity of Covid-19 and inflammatory processes (77). Therefore, S100B may serve as an important biomarker for neurocritical care disease detection and longitudinal monitoring in neurocritical care patients. S100B has been described as the brain’s CRP (C-reactive protein) due to its potential role as a neurological screening tool or biomarker of CNS injury, analogous to the role of CRP as a marker of systemic inflammation (78).

This meta-analysis has several strengths. First, this is the first systematic review and meta-analysis to evaluate the association between serum S100B levels and the risk of SAE and unfavorable prognosis in septic patients. Additionally, the sensitivity analysis results revealed that the pooled effect model was robust and reliable. There are also a few limitations to this meta-analysis should be taken care. First, our meta-analysis has potential publication bias, which may overstate the diagnosis and prognosis value of S100B. Second, the heterogeneity in our studies is high due to we cannot avoid confounding factors such as age, gender, sedation, hemodynamic status and primary diseases affecting the results. Third, although we aimed to comprehensively search the relevant articles, some studies may not have been published due to negative outcomes, and we do not have access to all the information for proper stratification analysis. In order to obtain better evidence, more high quality, prospective, multicenter randomized controlled studies with large sample sizes are needed.

## 5 Conclusion

Our Meta-analysis suggests that higher serum S100B level in septic patients is moderately associated with SAE and unfavorable outcomes (The outcomes here mainly refer to the death). Serum S100B may be a potential diagnostic and prognostic biomarker of SAE. Brian is an important target organ during sepsis, and our results provide ICU physicians with the most current information to predict which patients are at risk of SAE and take corresponding intervention measures to reduce morbidity and ameliorate neurological outcomes.

## Data availability statement

The original contributions presented in the study are included in the article/**Supplementary Material**. Further inquiries can be directed to the corresponding author.

## Author contributions

JH, SX, WL and LZ contributed to conception and design of the study. JH organized the database. JH and SX assessed the quality of the study. SX and WL performed the statistical analysis. JH wrote the first draft of the manuscript. JH and LZ wrote sections of the manuscript. All authors contributed to manuscript and approved the submitted version.
