# Supplementary material for: Diagnostic and prognostic value of serum S100B in sepsis-associated encephalopathy: A systematic review and meta-analysis
Source: Front Immunol. 2023 Jan 27;14:1102126. doi: 10.3389/fimmu.2023.1102126 (PMC9911439; doi:10.3389/fimmu.2023.1102126)
Supplement: Supplementary file 1 [file DataSheet_1.docx]

**Supplement Figure and Figure Legends:**


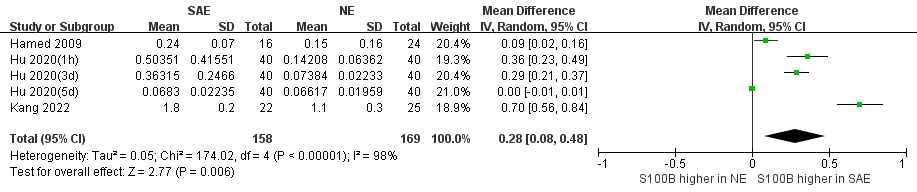


**Fig.S1.** Forest plot of comparison: association between serum S100B level and patients with SAE in children.


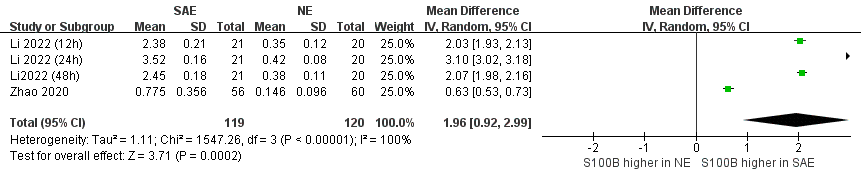


**Fig.S2.** Forest plot of comparison: association between serum S100B level and patients with SAE in burn paitients.
